# Taste Perception of Nutrients Found in Nutritional Supplements: A Review

**DOI:** 10.3390/nu11092050

**Published:** 2019-09-02

**Authors:** Thomas Delompré, Elisabeth Guichard, Loïc Briand, Christian Salles

**Affiliations:** CSGA (Centre des Sciences du Goût et de l’Alimentation), AgroSup Dijon, CNRS, INRA, Université de Bourgogne-Franche Comté, 21000 Dijon, France

**Keywords:** nutritional supplements, active compounds, taste, taste receptors, bitter

## Abstract

Nutritional supplements are prescribed when one’s nutritional status is not conducive to good health. These foodstuffs constitute concentrated sources of nutrients such as vitamins, minerals, amino acids, and fatty acids. For nutritional supplements to be effective, patients must consume the amount that has been prescribed for the recommended period of time. Therefore, special attention must be given to the sensory attributes of these products. Indeed, the presence of active compounds can cause an off-taste or aftertaste. These negative sensations can lead to a reduction in the consumption of nutritional supplements and reduce the effectiveness of the treatment. In this manuscript, we provide an overview of the sensory characteristics and the sensing receptor mechanism of the main compounds present in oral nutritional supplements, such as amino acids, minerals, fatty acids, and vitamins. Part of this article is devoted to the development of new masking strategies and the corresponding potential influence at the industrial level.

## 1. Introduction

Balanced and healthy food must meet our needs for nutrients. However, the many constraints of everyday life, undernutrition, and certain pathologies, such as liver and gastrointestinal diseases, cystic fibrosis, and certain cancers, are not conducive to maintaining a nutritional status [1,2]. Oral nutritional supplements can be prescribed to supply one or more nutrient deficiencies and restore the proper development and function of the body [3,4]. The aim of these foodstuffs is to supplement a normal diet and constitute a concentrated source of nutrients or other substances with a nutritional or physiological effect, alone or in combination [5]. A wide variety of supplements are available in pharmacy and drugstore market, and their formulation varies according to the target population (effervescent tablets, chewable, supplemented drinks, powders for oral use, or gelled). Oro-dispersible forms are sometimes characterized by negative perceptual sensations such as an off-taste or aftertaste. As the global flavor is a driver of acceptability by consumers, these off-tastes constitute an important technological barrier to consumer acceptance. It has therefore become essential for the manufacturers of these products to develop new strategies that are more inventive and effective to ensure acceptability. Only a small amount of data is available in the literature on the flavor properties of functional nutrients. Flavor perception is the functional integration of information transmitted by the chemical senses: olfaction, gustation and oral/nasal somatosensory inputs [6]. In the present review, the nutrients are non-volatile compounds and, as such, are not able to activate the olfactory receptors; thus, we will not detail the olfactive perception, including the smell perceived via the orthonasal route and the aroma perceived via the retronasal route. However, some off-flavors are due to volatile compounds formed by chemical reactions occurring during storage and manufacturing, these aspects will be briefly discussed in the manuscript. The present review will mainly focus on the taste perception of different types of pure nutrients used as supplements. The term taste will be used for the oral sensation perceived by the gustatory system [7]. The different basic taste modalities are sweet, sour, salty, bitter, and umami. Taste compounds also possess some trigeminal properties [8], mostly with astringent and irritant perceptions.

Indeed, dietary supplements are the results of the combinations of certain vitamins, minerals, amino acids, plant extracts, and polyunsaturated fatty acids for a combined and synergistic effect. Most of these nutrients have specific sensory properties, some of which are pleasant and some of which are unpleasant to the consumer. The first part of the manuscript will present an overview of the data available in the literature on taste, trigeminal descriptions, and the taste thresholds of the pure nutrients currently used in formulations, such as amino acids, fatty acids, minerals, and vitamins. Plant extracts will be excluded, as they are composed of a mixture of ingredients. These data will be compared with the amount of nutrients present in the formulations to predict their potential effect in the formulations. Specific attention will then be given to the sensory interactions occurring when these nutrients are used in mixtures, such as additive and masking effects. The last part of the manuscript will be devoted to the interactions between the nutrients and the food matrix and their effect to increases in the product acceptability and to the development of new strategies and their potential influence at the industrial level.

## 2. Taste of Amino Acids and Their Influence on Nutritional Supplement Taste

Free amino acids are usually consumed as nutritional supplements for a variety of reasons. Nine essential amino acids (l-histidine, l-isoleucine, l-leucine, l-lysine, l-methionine, l-phenylalanine, l-threonine, l-tryptophan, and l-valine) are not synthesized by the human body and need to be found in our food. The branched-chain amino acids (l-leucine, l-valine, l-isoleucine) are commonly used for athletic training and muscle repair [9,10], whereas essential amino acids such as l-tryptophan, l-phenylalanine and their metabolites seem to have the potential to improve energy, mood, and quality of sleep [11,12]. Non-essential amino acids such as l-glutamine are also commonly used as food supplements for their suspected benefit on digestion and the immune system [13].

**Figure 1:**
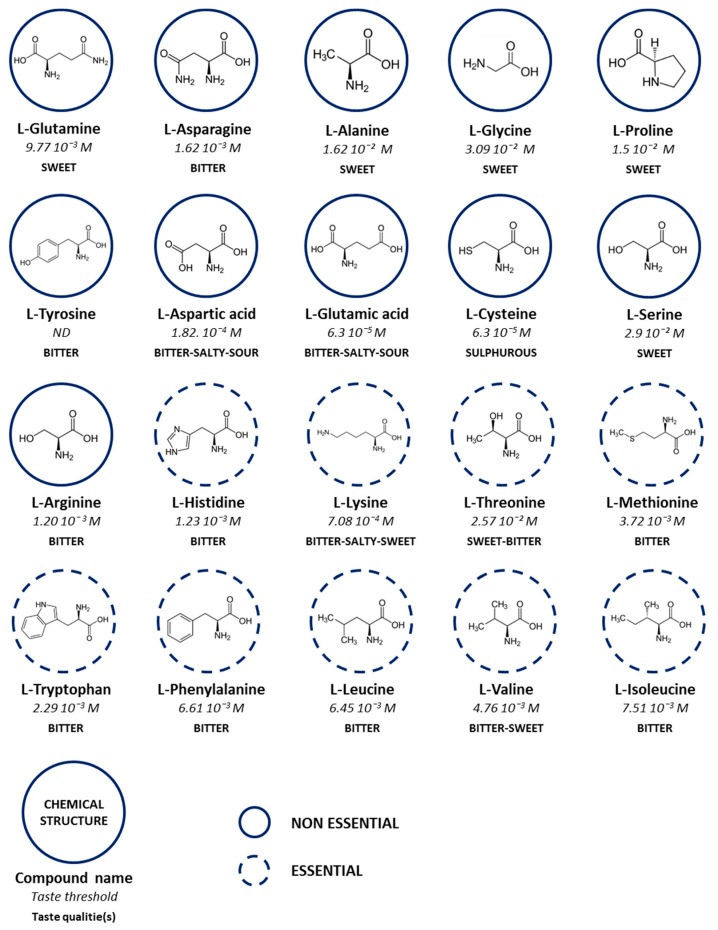
Structure, detection threshold values and taste qualities of the 20 l-amino acids [18]. Amino acids have been classified according to their availability for the body. ND, Not Determined.

The taste of free amino acids has been described long ago and is known to be very complex (Figure 1). Psychophysical experiments have shown that most amino acids and their salts have a taste. In addition, numerous amino acids elicit more than one of the five basic tastes: Sweet, umami (savoury), bitter, salty, and sour. When we observe the quantities used in different preparations available on the market, it is possible to affirm that the amino acids contribute to the sensory taste of powder with higher amino acid content. In most cases, detecting amino acids by the sensory sensing mechanism of taste is possible. Indeed, the threshold concentrations of 14 L-amino acids are lower than the L-amino acid concentrations in nutritional supplements (Table 1). In contrast, many of the gustatory receptors for the five basic tastes have been identified since the early 2000s. The tasting of compounds is mediated by these taste receptors expressed in taste receptor cells (TRCs) localized in the oral cavity [14]. The epithelial Na^+^ channel (ENaCs) and Otopretin1 (Otop1) are likely to be involved in transducing the salty and sour taste qualities [15,16], respectively. Umami, bitter, and sweet tastants are detected by G protein-coupled receptors (GPCRs). GPCRs are membrane proteins that share transduction mechanisms and structural features. The detection of bitter, sweet, and umami compounds involves a common transduction mechanism involving a heterotrimeric G protein including the Gα subunit named α-gustducin. When a tasting compound binds to a GPCR, intracellular signalling is activated, including α-gustducin signalling, leading to phospholipase C-β2 (PLC-β2) activation and an increase in inositol 1,4,5-triphosphate (IP_3_), which opens ion channels on the endoplasmic reticulum and releases Ca^2+^. The increase in the level of intracellular Ca^2+^ causes the transient potential ion channel of subfamily M member 5 (TRPM5) to open, which generates sodium influx and the depolarization of TRCs [17]. The contribution of taste receptors to amino acid sensing is becoming increasingly clear.

Three proteinogenic amino acids have been reported to elicit a sweet taste in humans, including l-glycine, l-alanine, and l-threonine. Interestingly, seven amino acids with the d configuration, (d-tryptophan, d-phenylalanine, d-leucine, d-histidine, d-isoleucine, and d-valine) elicit a sweet taste, while their l-forms do not exhibit a sweet taste [15]. These differences in the taste of the different enantiomeric forms can be explained by the activation of the sweet taste receptor by amino acids [15]. The sweet taste receptor is a heterodimer formed by the obligate assembly of two GPCRs named TAS1R2 (taste receptor type 1, member 2) and TAS1R3 (taste receptor type 1, member 3). The TAS1R2 and TAS1R3 subunits are members of the small family of class C GPCRs. This family includes the calcium-sensing receptor (CaSR), the metabotropic glutamate receptors (mGluR), and the metabotropic gamma-aminobutyric acid receptor (GABABR) [17]. Class C GPCRs share structural features, including an N-terminal domain (NTD) that is linked to the heptahelical transmembrane domain (TMD) by a cysteine-rich domain. The TAS1R2/TAS1R3 receptor [19] is able to detect a wide chemical variety of sweet tasting compounds, including carbohydrates (such as fructose, glucose, and sucrose) and natural (stevioside) and artificial (such as aspartame, saccharin, and cyclamate) sweeteners. The TAS1R2/TAS1R3 receptor is also activated by all of the sweet amino acids in both the l- and d configurations mentioned above, such as glycine, l-alanine and d-tryptophan [15,19]. The NTD of TAS1R2 contains the primary binding site of sweet compounds where d-glycine and d-tryptophan have been shown to interact [15]. Moreover, at least three other binding sites have been identified in this heterodimeric TAS1R2/TAS1R3 receptor. The presence of multiple binding sites in the sweet taste receptor causes the allosteric mechanism responsible for the synergy that is known to exist between some sweet tasting compounds.

Among the proteinogenic amino acids, l-glutamate and its salts (i.e., monosodium l-glutamate) are well known for their taste properties. l-glutamate is the prototypical stimulus that represents the umami taste quality (savoury). One feature of the umami taste is its potentiation by purinic ribonucleotides, such as guanosine-5′-monophosphate (GMP) and inosine-5′-monophosphate (IMP) [19,20,21]. The heterodimeric umami receptor is composed of two GPCRs named TAS1R1 (taste receptor type 1, member 1) and TAS1R3, common subunits in the sweet taste receptor. The TAS1R1/TAS1R3 receptor allows the detection of all umami tastants. Only two amino acids, l-glutamate and l-aspartate, have an umami taste for humans, whereas behavioral and electrophysiological experiments have revealed that rodents are able to perceive a large range of amino acids as umami tastants.

Numerous d and l amino acids can elicit a bitter taste. The bitterness of amino acids is often empirically related to their overall hydrophobicity. With regard to l amino acids, those with hydrophobic lateral chains, such as l-leucine, l-isoleucine, l-valine, l-arginine, l-methionine, l-phenylalanine, l-tyrosine, l-tryptophan, and l-histidine, exhibit a bitter taste [16]. Bitter compounds, including amino acids, are detected in the mouth by 25 different bitter taste receptors in humans, named TAS2Rs (taste receptor of type 2) [22]. In contrast, in addition to TAS1Rs, TAS2Rs belong to the very large family of class A GPCRs, which possess a short N-terminal domain. Molecular modelling and site-directed mutagenesis of TAS2Rs have demonstrated that the ligand binding site is located within the TMD. *In vitro* binding assays have revealed the binding profile of 20 out of the 25 human bitter taste TAS2Rs. It has been shown that some TAS2Rs detect only a few bitter molecules, whereas others are broadly tuned to detect numerous bitter compounds [23]. To date, 4 TAS2Rs have been qualified as orphan receptors; that is no bitter compounds that are capable of activating them have been identified. The activation of bitter TAS2Rs is responsible for the bitter off-taste of some compounds, such as the unwanted aftertaste of some sweeteners such as saccharin and stevioside. Cellular assays have shown that the detection of bitter amino acids is due to the activation of a set of five TAS2R receptors [24]: TAS2R1, TAS2R4, TAS2R8, TAS2R39, and TAS2R43. For instance, the authors of this study observed that L-phenylalanine and L-tryptophan could activate TAS2R1 and TAS2R4, respectively, whereas TAS2R4 and TAS2R39 both responded to d-tryptophan (Table 2).

In addition to the five basic tastes, a taste sensation named kokumi was proposed approximately 28 years ago [25]. In Japanese, kokumi means “mouthfullness and thickness”. Kokumi molecules have no taste themselves, but they are able to enhance the sweet, umami, and salty tastes. Tripeptide glutathione (Glu-Cys-Gly) is the prototypical kokumi taste compound. GSH is tasteless, but in the presence of umami compounds, it reinforces their taste and increases the long-lasting taste sensation [25]. It has been shown that kokumi taste detection involves the calcium-sensing receptor (CaSR), which is expressed in TRCs [26]. Interestingly, cellular assays have revealed that some amino acids, such as l-histidine, l-tryptophan, l-phenylalanine, and l-tyrosine, can moderately activate the CaSR receptor and produce a kokumi taste in humans [27]. Although the sensorial impacts of the amino acids thought to be involved in the kokumi taste are poorly understood, we can speculate that the involvement of the CaSR receptor in taste perception causes the taste interactions between some more complex amino acids.

## 3. The Taste of Polyunsaturated Fatty Acids and Their Influence on Nutritional Supplement Taste

Polyunsaturated fatty acids (PUFAs) are used as nutritional supplements for balancing blood lipid levels and preventing or reducing the risk of developing atherosclerotic changes, disorders and diseases. Fish oil is an important food source of PUFAs, has 18, 20 or 22 carbon atoms and is classified as an omega-3 or omega-6 fatty acid. The main problem with the use of fish oil as a nutritional supplement is the residual fishy odor that arises from the oxidation of the unsaturated bonds, leading to the generation of volatile odorant compounds. The major components of dietary fats are triglycerides, but the orosensory effective stimuli come from fatty acids. A positive correlation has been found between lipolysis activity and fat intensity [28], which confirms the hypothesis that fatty acids are released in the saliva by the hydrolysis of triglycerides and that fatty acids are detected in the oral cavity. Therefore, it is difficult to calculate the amount of fatty acids present in nutritional supplements and their impact on flavor perception.

To determine the real taste contribution of fatty acids, sensory tests were performed with a nose clip and the addition of texturing agents to avoid olfactory perception and textural differences. Limited data are available on the taste properties of polyunsaturated fatty acids, but some information is available on the taste detection thresholds related to C18 unsaturated fatty acids (Table 3). Concerning C20 and C22 PUFAs, such as eicosapentaenoic acid (EPA, C20:5, *n*-3) and docosahexaenoic acid (DHA, C22:6, *n*-3), only data on their effects on other taste perceptions are available.

Fatty acids are not perceived as fatty; all of them are described as irritants, and some of them are also perceived as metallic (stearic, linoleic, and linolenic acids), bitter (stearic, oleic, linoleic, and linolenic acids), astringent (oleic and linoleic acids), or nutty (oleic acid). A comparison of the taste thresholds calculated for saturated fatty acids [29] shows that the value increases with the fatty acid carbon chain length. This trend was observed for caproic (C6:0), lauric (C12:0), and oleic acid (C18:1), for which the taste detection thresholds measured with a nose clip were 1.45 mM, 5.37 mM, and 25.7 mM [33], respectively. The authors explained that FFAs with a shorter chain length are more soluble in water and thus allow easier access to the taste receptors. Concerning PUFAs, the mean taste thresholds for linoleic (C18:2) and α-linolenic acids (C18:3) were 5.6 and 2.5 times lower, respectively, than that for oleic acid (C18:1) [32]. These differences could not be explained by differences in either viscosity or particle size. High degrees of unsaturation lead to increased solubility and high diffusion rates across cell membranes or a different affinity for the receptor. The values found in the literature for these PUFAs are on the same order of magnitude, that is between 1.21 and 3.15 mM for linoleic acid (C18:2) and 3.15 mM for alpha-linolenic acid (C18:3, *n*-3). More data are available on the taste threshold for oleic acid (C18:1) with great variability among the authors, ranging from 2.2 to 25.7 mM for the detection thresholds measured with nose clip and from 0.78 to 2.23 mM for the detection thresholds measured without a nose clip. The lower thresholds measured without a nose clip can be explained by the presence of trace amounts of volatile odorants formed by the oxidation of unsaturated fatty acids.

Despite the differences in the medium detection threshold values, great differences exist between subjects. These inter-individual differences may be related to differences in salivary lipolytic activity [36,37]. A positive correlation has been found between lipolytic activity and FFA concentration in saliva, suggesting that lipolytic activity is responsible for the formation of free fatty acids from the endogenous salivary esterified fatty acids. Thus, a high salivary lipolytic activity will produce a high amount of free fatty acids in saliva, which could induce a high taste threshold for free fatty acids, due to an adaptation of the taste receptors to the basal salivary concentration in fatty acids as proposed for oleic acid [38]. It has also been shown that the orosensory threshold for triolein and oleic acid decreases after lipolytic activity inhibition [38], which confirms the role of salivary composition in orosensory perception. These inter-individual differences in the taste detection thresholds to fatty acids may influence the consumption of fat, as this result was observed by different authors. Participants who were more sensitive to fat (low thresholds) preferred low fat concentrations [34] and tended to consume a small amount of high-fat foods [35,39]. Moreover, it seems that the consumption of a low-fat diet increased the subject’s taste sensitivity to oleic acid [40] and their ability to perceive small differences in the fat content of custard.

The existence of fat taste detectors in humans is still under debate [41]. Different types of lipid sensors have been proposed to be involved in the chemoreception of fatty acids. While the role of two GPCRs (GPR40 and GPR120), which have been reported as potential fatty acid receptors in rodents, are uncertain, the gustatory function of Cluster of Differentiation 36 (CD36), a transporter/receptor belonging to the class B scavenger receptor family, has been extensively studied in humans. This transmembrane glycoprotein is expressed in rodent and human taste buds and binds long-chain fatty acids with a high affinity, and it has been observed in humans that the CD36 genotype affects the orosensory detection of fat [38,42,43]. In addition, it has been shown that subjects homozygous for the rs1761667 G-allele (high CD36 expression) have lower detection thresholds for oleic acid and triolein than subjects that are homozygous for the A-allele (low CD36 expression) [44].

## 4. Taste of Minerals and Their Influence on Nutritional Supplement Taste

Minerals are essential nutrients that our body needs in small amounts to work and keep us in good health. They are particularly necessary for building strong bones and teeth, controlling body fluids inside and outside of cells, and turning food into energy [45]. Minerals are provided mainly by foods and drinks, such as meat, cereals, fish, milk, dairy foods, fruits, vegetables, and mineral water; no single food item contains all of the required minerals, and thus, a single food item cannot provide all required minerals. A healthy balanced diet should provide all of the minerals to avoid diet deficiencies, but changes in modern dietary habits contribute to an important reduction in mineral nutrient intake levels. Macrominerals-sodium, potassium, calcium, magnesium, chloride, and sulphur—are essential mineral nutrients. Other minerals, such as iron, manganese, copper, iodine, zinc, cobalt, fluoride, and selenium, are considered trace elements because they are needed in smaller amounts [45]. However, these trace elements may be essential. For example, iron is involved in a wide variety of metabolic processes, and its deficiency leads to chronic diseases [46]. Most mineral salts have taste properties, but some, such as zinc and copper, are necessary to maintain normal chemoreception. The taste properties of mineral salts, including the threshold values, are presented in Table 4 and Table 5. It appears that many mineral salts are involved in taste perception. Threshold detection has been reported for most of the mineral salts studied; however, their values are dispersed over a large range according to not only the type of salt but also the same salt, and they vary significantly according to the type of water in which the salt is dissolved. Unfortunately, very little information is available concerning the taste properties of minerals, which may be complex. It is difficult to estimate the impact of minerals on the sensory taste of nutritional supplements. Although the quantities to be used are indicated on the pack, mineral salts incorporated in the preparation are concealed by the pharmaceutical industries. However, the patents available in the scientific literature and the current research suggest that some mineral salts are involved in the taste of nutritional supplements.

Sodium chloride is a well-known salting agent that is extensively used in everyday life. However, other mineral and organic salts have been characterized by a lower salt intensity than NaCl and various taste qualities [52]. In particular, the authors reported that at concentrations that produce a similar total intensity, all of the chloride salts except calcium chloride were saltier than their nonhalide counterparts, and the organic salts were considerably less salty than the inorganic salts. Salts with heavier cations, such as potassium and calcium, were also more bitter than the corresponding sodium salts. The use of potassium chloride is an alternative sodium substitute in food products. Potassium is an essential mineral nutrient that plays an important role in human organism functioning, particularly by contributing to the prevention of high blood pressure. However, this replacement introduces bitter and metallic off-tastes that affect consumer acceptance.

Calcium salts are currently used as nutritional fortifying agents but have complex and unpleasant flavor properties such as bitterness, sourness, astringency, and a metallic perception, with the predominance of bitterness. Among calcium salts, calcium chloride, which presents a salty taste at medium and high concentrations, is characterized by a notable bitter taste. The suppression of the unpleasant taste properties of calcium chloride can be obtained by substituting the chloride anion with a gluconate, glycerophosphate, or lactate anion [53].

Zinc salts are added to most nutritional supplements. The perception intensity of zinc salts (i.e., zinc bromide, sulphate, iodide, acetate, and chloride) was found to be very weak for bitterness, saltiness, savoriness, sourness and tingliness [54]. Only sourness was reported for zinc iodide but this result is possibly due to confusion with astringency and the carry-over effect. The major sensation elicited by zinc salts is astringency [54]. Zinc ions may bind to salivary proteins, resulting in a change in their structure and a reduction in salivary lubrication. However, the nature of the associated anion modulates the astringency intensity. Zinc iodide was significantly more astringent than zinc acetate, sulphate, and bromide. Thus, a careful choice of the anion in the formulation of nutritional supplements may limit this off-flavor perception. Compared to the other evaluated sensations, the astringency of the zinc salts was not significantly reduced after two oral rinses, thus showing an important lingering effect. This effect may be due to the binding of zinc ions to epithelial proteins with a strong enough affinity to persist after rinsing with pure water [54].

Mineral ions such as iron and copper may be involved indirectly in metallic flavor perception, which is defined as a combination of taste and retronasal odor [55]. These authors suggested that the production of the metallic flavor is due to salivary protein oxidation by minerals and the production of oxidation-related aldehydes related to odorant lipids. The metallic sensation due to stimulation with ferrous sulphate solutions was found to be suppressed when the nose was occluded, showing that this perception was fully due to the development of a retronasal smell [56]. This phenomenon was not observed for copper and zinc sulphates, which were found to be more bitter and astringent, respectively, and less metallic. Iron salts also exhibited other sensations, such as bitterness, sourness and astringency, which differ in their predominance according to the associated counteranion. These temporal sensory properties have been explored with ferrous sulphate, chloride, and gluconate [57]. The authors reported that these three compounds exhibited strong and persistent metallic flavors, but the oral sensations were complex and changed with time. Ferrous chloride had the most bitter taste and ferrous gluconate had the highest intensity of initial sourness and astringency. Taste properties were found to decay rapidly; although predominant sensations change over time, the long-term lingering of astringency and metallic tastes may limit the use of simple iron salts in supplement formulations [57]. The threshold values of iron and copper sulphates and chlorides were compared with two modalities: Without a nose clip and with a nose clip (Table 4); in each case, smaller threshold values were observed without the nose clip than with the nose clip [50], thus demonstrating the retronasal olfactory perception dimension of these minerals. Concerning copper sulphate and chloride, it has been reported that the perception threshold value does not change significantly when the nose is either clamped or not clamped [58]. However, the perception threshold is dependent upon the quality of the water in which the salts are dissolved (Table 4 and Table 5); this result has been reported for several divalent metallic salts, where the threshold value in distilled water was found to be lower than that in the same amount of spring water.

## 5. Taste of Vitamins and Their Influence on Nutritional Supplement Taste

**Figure 2:**
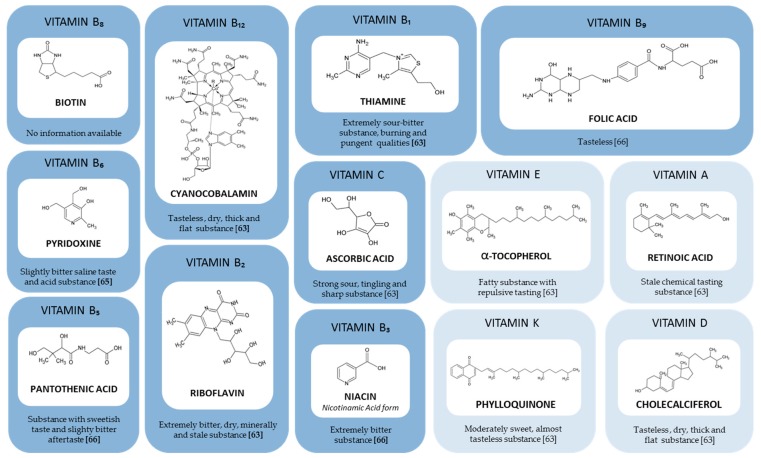
Chemical structures and taste qualities of 13 vitamins. Vitamins have been classified according to their solubility. 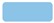 water-soluble vitamins, 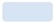 fat-soluble vitamins.

Vitamins are substances without energetic value but are essential for correct human body functions. There are currently thirteen vitamins divided into two groups. First, there are nine water-soluble vitamins: B1 (thiamine), B2 (riboflavin), B3 (niacin), B5 (pantothenic acid), B6 (pyridoxine), B8 (biotin), B9 (folic acid), B12 (cobalamin), and C (ascorbic acid) (Figure 2). With the exception of cobalamin, these vitamins are not stored for prolonged periods of time in the body, and their excess is excreted in urine [59]. Second, there are four fat-soluble vitamins: A (retinoic acid), D (calciferol), E (tocopherol), and K (phylloquinone). These vitamins are assimilated at the same time as lipids during digestion and are stored in fat tissue.

With the exception of two vitamins (K and D), the human body is unable to produce vitamins. As a result, vitamin intake from food is essential for correct body function. These substances are involved in a large number of physiological processes: Coenzyme function, electron and proton transport, membrane stabilization, and hormonal and gustatory function. For example, vitamin K is essential for maintaining an optimal level of certain coagulation factors, whereas ascorbic acid is a great antioxidant [60]. It has generally been accepted that a balanced diet provides a necessary quantity of vitamins. However, certain pathologies, certain drug therapies and limited access to balanced nutrition may lead to vitamin deficiencies [61]. Since the recognition of vitamins as a requirement in our diet and the identification of vitamin deficiencies, many pharmaceutical preparations have been manufactured by pharmaceutical companies in several forms, such as tablets, capsules, and liquid mixtures for oral use. The main problem with their use as nutritional supplements is their negative perceptual sensations, such as an off-taste.

Unlike the different active ingredients found in oral nutritional supplements, the taste quality of vitamins and their impact on the sensory perception of nutritional supplements have been poorly studied. Indeed, only a limited amount of recent data are available in the scientific literature. Although no information is available on the detection thresholds of these vitamins, some databases, books and scientific articles describe their organoleptic qualities (Figure 2). In 1949, certain vitamins, specifically the vitamin B complex, were already identified as being involved in the off-taste in some pharmaceutical preparations [62]. Years later, in 1975, the first descriptive sensory analysis of 8 vitamins, some belonging to the vitamin B complex (B1, B2, and B12), was carried out [63]. The sensory analyses were performed with a sensory panel of experts using nose clips to avoid olfactory perception. Vitamins B1 and B2 were considered to be extremely bitter and unpleasant by all panelists. *In vitro* cellular assays have demonstrated that vitamin B1 activates three bitter taste receptors, TAS2R1, TAS2R7 and TAS2R39 [22,64]. The activation of these receptors was not be demonstrated after the addition of vitamin B2 with the different TAS2Rs. Vitamin B1 has also been described as burning and pungent, which are two trigeminal sensations. Of the 8 active ingredients tested, vitamins A, B12, D3 and K1 have been evaluated as tasteless. In contrast, vitamin E has been characterized as fat and repulsive with an unpleasant taste, while vitamin C has been described as sour and fruity, with a pleasant taste for half of the judges and an unpleasant taste for the other half. This first and only sensory study on the taste of certain vitamins provides us with some information on their sensory quality and informs us about their possible implications in the off-tastes of certain pharmaceutical preparations. The untested vitamins (B3, B5, B6, B8, and B9) from this first screening by Schiffman are not tasteless [63]. Although no other sensory study has been performed on these vitamins, information about their organoleptic qualities can be found in books and online databases. Thus, it is possible to conclude the bitterness and acidity of pyridoxine hydrochloride [65], the low bitterness of vitamin B5 analogues, calcium pantothenate and pantothenol [66], and the sensation of bitterness and salinity caused by the ingestion of vitamin B3 [66]. These few scientific books and online databases allow us to broaden and confirm the conclusions formed previously.

## 6. Interactions Between Nutrients of Nutritional Supplements

As described above, most of the nutrients contained in nutritional supplements have specific taste qualities. In a mixture of nutritional supplements, sensory interactions can be observed. Several interactions are possible when taste compounds are mixed. Enhancement results when the effect of the addition of one compound to another on a taste attribute is higher than the sum of the individual effects of the two compounds; additivity occurs when this sum corresponds to the sum of the individual effects of the two compounds; and suppression or a masking effect occurs when this sum is less than the individual effects of the two compounds [67]. In nutritional supplements, several cases of sensory interactions have been reported.

The masking effects of mineral cations on taste perception occur in mammalian species. As an example, divalent heavy mineral cations were reported to have a masking effect on all taste stimuli (bitter, sour, sweet, and salty), while copper and zinc chlorides only masked the response to sweet stimuli in mice [68]. These authors reported that copper and zinc chloride (10^−5^ M) masked the sweet response to sucrose and sodium saccharin without affecting the other taste responses, while the presence of iron sulphate, manganese, cobalt, nickel or cadmium chlorides had no effect on the sweet response or only slightly masked it at high concentrations.

Zinc (tested as the sulphate salt) was found to alter the sweetness and bitterness perceptions but did not affect saltiness, savoriness or sourness perceptions [54]. Zinc masked the sweetness of glucose at all concentrations, suggesting a non-competitive suppression mode, but such masking effect was not observed with magnesium sulphate. Thus, zinc is a potent masking agent of sweetness in humans. Concerning the inhibition of bitterness, sodium and zinc salts were found to mask the bitterness of certain compounds through action at the peripheral level [69]. It has been shown that zinc sulphate masks the bitterness of bitter compounds such as quinine at all concentrations tested [54] or tetralone and denatonium benzoate, whereas it has been shown to be ineffective on the bitterness of sucrose octa-acetate, pseudo ephedrine chlorhydrate, and dextromethorphan [70]. In particular, zinc lactate significantly masked the bitterness of 3 and 4 mM caffeine but not 1.5 mM caffeine. The authors suggested that the zinc ions allosterically modulate the transmembrane GPCR receptors, presumably the TAS2Rs. However, the potential use of zinc ions to mask bitterness in foods and pharmaceuticals is limited because zinc ions may also mask sweetness [71]. Sodium gluconate was found to mask the bitterness of caffeine but to a lesser extent than zinc ions. After the effect of different salts on different bitter compounds was studied, it was reported that some salts interact with bitter compounds differently than other salts [70]. As example, zinc sulphate, sodium acetate, and magnesium acetate significantly suppressed the bitterness of bitter compound. The addition of zinc lactate to products containing caffeine, such as coffee and chocolate, was found to significantly reduce bitterness [69]. Despite the bitterness inhibition properties of zinc, its potential use as a bitterness masking agent in supplement formulations is limited because of its potent sweetness masking properties. However, zinc can be used with sodium cyclamate as a sweetener in bitter–sweet formulations because zinc sulphate does not mask the sweetness of sodium cyclamate. The bitterness suppression by zinc ions is suggested to originate from interactions with the bitter taste receptors at the peripheral level through the formation a complex with the extracellular portion of the TAS2Rs, whereas the bitterness masking effect by the sweetness perception is suggested to originate from central-level mechanisms. These hypotheses remain to be confirmed by experimental data.

Sodium chloride is an effective bitterness inhibitor [72]. By testing several combinations of cations and anions, these authors reported that the sodium cation inhibited the bitterness of pharmaceuticals more than the other tested cations, such as potassium, calcium, magnesium, and ammonium, which inhibited bitterness at lower, varying degrees. Anions were also found to inhibit bitterness to various degrees when associated with the sodium cation, but the glutamate and adenosine monophosphate anions were more efficient compared to other anions such as chloride, gluconate, and glycerophosphate [72]. Although sodium chloride is a masking agent of some bitter compounds, such as calcium chloride [67], some exceptions exist; for example, the bitterness of magnesium chloride is not masked by sodium chloride. Moreover, the intrinsic salty taste of calcium chloride is additive to the salty taste of sodium chloride. A single, broad bitter taste receptor that is able to detect a broad range of bitter divalent and trivalent salts has recently been identified [73,74]. Using a cell-based functional assay, two studies have revealed that the bitter taste receptor TAS2R7 is responsible for the detection of a broad range of minerals, including zinc, calcium, magnesium, iron, copper, manganese, and aluminium, but not potassium ions.

Concerning calcium chloride, its bitterness can be suppressed by using masking agents. As an example, sucrose was found to suppress the bitterness of calcium chloride, and citric acid was found to suppress the bitterness at high levels of calcium chloride, with a slight suppressive effect on the metallic perception [75]. Additionally, sucrose was found to decrease sourness, and citric acid was found to decrease sweetness. The addition of sugars such as sucrose or trehalose decreases the bitter and metallic perceptions of potassium chloride and thus enhances the saltiness perception and the sweetness perception [53].

Fatty acids also contribute to the modulation of taste perception. Linolenic acid, EPA, and DHA were shown to substantially increase the umami taste intensity and decrease bitterness [76]. Moreover, an increase in the content of DHA in oil tends to reduce bitterness and increase the umami perception in both model emulsions and a synthetic tuna extract emulsion [77].

Concerning vitamins, although vitamins such as B3, B5, and B6 may cause negative perceptual sensations, some may have a positive impact on sensory perception by acting as inhibitors of some tastes such as bitterness [78]. For example, a sensory study conducted in Japan demonstrated that a vitamin B5 derivative, calcium pantothenate (0.01 and 0.02%), was able to reduce the bitterness generated by the ingestion of saccharin or caffeine.

Such masking effects of some components could be used advantageously by industries as a strategy to avoid off-tastes in the formulation of nutritional supplements.

## 7. Conclusion and Perspectives

Nutritional supplements are mixtures of ingredients, most of which have taste properties. For some of these components, such as vitamins and some specific minerals, essential data such as perception threshold values and taste qualities are missing and need to be considered in the development of the product. However, for most of the components used in the formulation of nutrients, notes of bitterness or metal are not preferred by consumers and may result in a rejection of the product and a negative economic impact. Therefore, flavoring is a crucial aspect to consider in improving the formulation of supplements. The main objective of flavoring supplements is to increase their acceptability by the users, particularly with the masking of off-flavors.

The extended-release formulations based on the use of orally disintegrated capsules and hot melt coating are a challenge in tuning the release profile to meet the needs of the product, but they can also deliver a pleasant oral experience by offering an enhanced and prolonged taste, aroma and mouthfeel that modern consumers have come to expect. Thus, a potential strategy may be taking advantage of the development of new excipients and integrating their potential masking properties of bitterness or undesirable aromatic notes into the selection criteria [79]. For example, fishy burps are a challenge in delivering odorous oils such as omega-3 in nutritional supplements. Now, new microemulsion technologies have been explored as innovative and revolutionary solutions, leading to reduced off-flavors and increased acceptability by consumers.

In this review, we have reported that in the mixture context of taste compounds, sensory interactions occur that can lead to additive or masking effects. These taste-taste interactions are dependent on the concentration of the taste stimuli. Particularly, it has been reported that bitterness can be suppressed by saltiness at subthreshold concentration, by sweetness, sourness, and saltiness at suprathreshold concentration, and by sweetness and saltiness at higher stimuli concentration [67]. Therefore, these interactions may be another tool in masking bitterness in nutritional supplements.

The physiological mechanism involved in taste-taste interactions are not yet well understood, but they are thought to occur at the taste receptor cell level. A thorough understanding of the mechanisms involved in taste receptors is therefore necessary to be able to fully utilize the potential of the interactions between bitterness and other tastes and to identify molecules blocking the perception of bitterness.

Lastly, it is also noteworthy that specific aromas are known to be able to enhance taste intensity such as sweetness and saltiness intensities, through cognitive interactions, but specific aroma can also suppress taste [80]. Thus, it could also be possible to use aromas to limit undesirable tastes such as bitterness in supplements by using, for example, sweetness-associated aromas.

## Figures and Tables

**Table 1:** Comparison between threshold concentrations and higher levels of l-amino acids concentrations in 22 g of effervescent powder dissolved in a volume of 200 mL of water. This powder is a unique complex of amino acids from pure protein sources, whey protein, and calcium caseinates (Nutritional Technologies International, Optizone ™).

Amino Acids	Threshold Concentration (mol/L)		Amino Acids Concentration (mol/L)
l-Alanine	1.62 × 10^−2^	<	1.72 × 10^−2^
l-Arginine	1.20 × 10^−3^	<	4.31 × 10^−3^
l-Aspartic acid	1.82 × 10^−4^	<	2.55 × 10^−2^
l-Cysteine	6.3 × 10^−5^	<	5.5 × 10^−3^
l-Glutamic acid	6.3 × 10^−5^	<	4.1 × 10^−2^
l-Glycine	3.09 × 10^−2^	>	6.3 × 10^−3^
l-Histidine	1.13 × 10^−3^	<	3.85 × 10^−3^
l-Isoleucine	7.51 × 10^−3^	<	1.5 × 10^−2^
l-Leucine	6.45 × 10^−3^	<	2.58 × 10^−2^
l-Lysine	7.08 × 10^−4^	<	2.07 × 10^−2^
l-Methionine	3.72 × 10^−3^	<	4.99 × 10^−3^
l-Phenylalanine	6.61 × 10^−3^	<	6.4 × 10^−3^
l-Proline	1.5 × 10^−2^	<	1.73 × 10^−2^
l-Serine	2.9 × 10^−2^	>	1.46 × 10^−2^
l-Threonine	2.57 × 10^−3^	<	1.72 × 10^−2^
l-Tryptophan	2.29 × 10^−3^	>	2.19 × 10^−3^
l-Valine	4.76 × 10^−3^	<	1.63 × 10^−2^

<, threshold concentration is lower than the amino acids concentration; >, threshold concentration is higher than the amino acids concentration.

**Table 2:** Response profile of six TAS2Rs to d- and l-forms of tryptophan [15,24] and phenylalanine [15].

	TAS2R1	TAS2R4	TAS2R8	TAS2R39	TAS2R43	TAS2R49
l-Phenylalanine	+	+	+	+	−	−
l-Tryptophan	+	+	−	+	+	+
d-Phenylalanine	+	−	−	+	−	−
d-Tryptophan	−	+	−	+	−	−

+, response; −, no response.

**Table 3:** Taste qualities and threshold concentration of fatty acids in different medium with or without nose-clip.

Formula	Name	Taste Qualities	Threshold Concentration (M)	Medium
C_12_H_24_O_2_	Lauric acid	ND	0.035 × 10^−3^ [29]	X
C_14_H_28_O_2_	Myristic acid	ND	0.22 × 10^−3^ [29]	X
C_16_H_32_O_2_	Palmitic acid	ND	3.9 × 10^−5^ [29]	X
C_18_H_36_O_2_	Stearic acid	Irritant, metallic, bitter	1.4 × 10^−5^ [30]	UHT milk 3.5% fat
		ND	0.01 × 10^−3^ [31]	Water, 5% gum acacia
		ND	0.05 × 10^−3^ [29]	Emulsion, 5% gum acacia
C_18_H_34_O_2_	Oleic acid	ND	* 1.99 × 10^−2^ [32]	10% gum arabic, 0.05% xanthan
		Irritant, bitter, sour	* 2.57 × 10^−2^ [33]	10% gum arabic, 0.05% xanthan
		Astringent, bitter	3.9 × 10^−3^ [30]	UHT milk 3.5% fat
		ND	2.0 × 10^−2^ [34]	Skim milk 5% gum acacia
		ND	* 2.2 × 10^−3^ [35]	Non-fat milk
		ND	* 3.5 × 10^−3^ [36]	10% skimmed milk powder
		ND	2.23 × 10^−3^ [36]	10% skimmed milk powder
		ND	0.78 × 10^−3^ [31]	Emulsion, 5% oil, 5% gum acacia
		ND	0.28 × 10^−3^ [29]	X
C_18_H_32_O_2_	Linoleic acid	Metallic, astringent, bitter	2.39 × 10^−3^ [30]	UHT milk 3.5% fat
		ND	* 1.5 × 10^−3^ [35]	Non-fat milk
		ND	1.21 × 10^−3^ [31]	Emulsion, 5% oil, 5% gum acacia
		ND	1.55 × 10^−3^ [32]	10% gum arabic, 0.05% xanthan
		ND	0.039 × 10^−3^ [29]	X
C_18_H_30_O_2_	Alpha-linolenic acid	ND	* 3.15 × 10^−3^ [32]	10% gum arabic, 0.05% xanthan
		Irritant, metallic, bitter	0.41 × 10^−3^ [30]	UHT milk 3.5% fat

ND, Not Determined; *, with nose clip; X, not specified.

**Table 4:** Taste qualities and threshold concentration of mineral salts in different mediums, with or without nose-clip.

Formula	Name	Taste Qualities	Threshold Concentration (M)	Medium
NaCl	Sodium chloride	Salty	* 8.0 × 10^−3^ [29]	Water
KCl	Potassium chloride	Salty, bitter, metallic	* 1.7 × 10^−2^ [29]	Water
CaCl_2_	Calcium chloride	Bitter, salty	* 1.0 × 10^−2^ [29]	Water
MgCl_2_	Magnesium chloride	Bitter	* 1.5 × 10^−2^ [29]	Water
MgSO_4_	Magnesium sulphate	ND	* 4.6 × 10^−3^ [29]	Water
LiCl_2_	Lithium chloride	Salty, sour	* 2.5 × 10^−2^ [29]	Water
NaI	Sodium iodide	ND	* 2.8 × 10^−2^ [29]	Water
CuSO_4_	Copper sulphate	ND	* 6.2 × 10^−6^ [47]	Water
Na_2_SO_4_	Sodium sulphate	Salty, bitter	* 1.7 × 10^−3^ [48]	Water
CaSO_4_	Calcium sulphate	Salty, bitter	* 8.3 × 10^−4^ [48]	Water
Na_2_NO_3_	Sodium nitrate	Untasty	* 1.6 × 10^−3^ [48]	Water
CaNO_3_	Calcium nitrate	Untasty	* 1.6 × 10^−3^ [48]	Water
FeSO_4_	Ferrous sulphate	ND	* 9.9 × 10^−5^ [49]	Deionized water
FeSO_4_	Ferrous sulphate	Metallic	* 3.0 × 10^−5^ [50]	Deionized water
FeSO_4_	Ferrous sulphate	ND	1.6 × 10^−4^ [50]	Deionized water
FeCl_2_	Ferrous chloride	ND	* 6.6 × 10^−5^ [49]	Deionized water
FeCl_2_	Ferrous chloride	ND	* 6.4 × 10^−5^ [50]	Deionized water
FeCl_2_	Ferrous chloride	ND	2.27 × 10^−4^ [50]	Deionized water
CuSO_4_	Copper sulphate	Bitter, astringent, metallic	* 7.8 × 10^−6^ [50]	Deionized water
CuSO_4_	Copper sulphate	Bitter, astringent, metallic	2.46 × 10^−5^ [50]	Deionized water
CuCl_2_	Copper chloride	ND	* 8.2 × 10^−6^ [50]	Deionized water
CuCl_2_	Copper chloride	ND	1.56 × 10^−5^ [50]	Deionized water

ND, Not Determined; *, without nose clip.

**Table 5:** Taste qualities and threshold of cations (mol cation/L) in different medium with or without nose-clip [51].

Formula	Name	Taste Qualities	Threshold Concentration (mol cation/L)	Medium
ZnSO_4_	Zinc sulphate	ND	* 4.12 × 10^−4^	Spring water
ZnSO_4_	Zinc sulphate	ND	* 2.75 × 10^−4^	Distilled water
ZnNO_3_	Zinc nitrate	ND	* 3.36 × 10^−4^	Distilled water
ZnCl_2_	Zinc chloride	ND	* 4.12 × 10^−4^	Spring water
ZnCl_2_	Zinc chloride	ND	* 5.04 × 10^−4^	Distilled water
CuCl_2_	Copper chloride	ND	* 2.04 × 10^−4^	Spring water
CuCl_2_	Copper chloride	ND	* 1.03 × 10^−4^	Distilled water
FeSO_4_	Ferrous sulphate	ND	* 3.21 × 10^−5^	Spring water
FeSO_4_	Ferrous sulphate	ND	* 6.07 × 10^−5^	Distilled water
Fe_2_H_2_0_4_	Hydrous ferric oxide	ND	* 1.57 × 10^−4^	Distilled water
MnSO_4_	Manganese sulphate	ND	* 8.19 × 10^−4^	Distilled water
CuSO_4_	Copper sulphate	ND	* 3.78 × 10^−5^	Distilled deionized water
CuSO_4_	Copper sulphate	ND	* 5.51 × 10^−5^	Uncarbonated water
CuCl_2_	Copper chloride	ND	* 3.94 × 10^−5^	Distilled deionized water
CuCl_2_	Copper chloride	ND	* 5.98 × 10^−5^	Uncarbonated water

ND, Not Determined; *, without nose clip.
